# Regulation of T cell exhaustion and stemness: molecular mechanisms and implications for cancer immunotherapy

**DOI:** 10.1038/s41423-025-01378-4

**Published:** 2025-12-19

**Authors:** Zeyu Chen, Ziang Zhu, Taidou Hu, Chen Yao, Tuoqi Wu

**Affiliations:** 1 Department of Cancer Biology, Dana-Farber Cancer Institute, Boston, MA USA; 2 Gene Regulation Observatory, Broad Institute, Cambridge, MA USA; 3 Department of Cell Biology and Pathology, Harvard Medical School, Boston, MA USA; 4 Department of Immunology, University of Texas Southwestern Medical Center, Dallas, TX USA; 5 Immunology Ph.D. Program, University of Texas Southwestern Medical Center, Dallas, TX USA; 6 Simmons Comprehensive Cancer Center, University of Texas Southwestern Medical Center, Dallas, TX USA; 7 Kidney Cancer Program, Simmons Comprehensive Cancer Center, University of Texas Southwestern Medical Center, Dallas, TX USA; 8 Peter O’Donnell Jr. Brain Institute, Dallas, TX USA

**Keywords:** T cell exhaustion, Stem-like T cells, Immunotherapy, Cancer immunology, Chronic infection, Lymphocyte differentiation, Immunotherapy

## Abstract

T cells are central components of the adaptive immune system and play key roles in antitumor and antiviral responses. The diverse cell fates of T cells enable them to respond to different durations and strengths of antigen stimulation and various cytokine milieus in a context-dependent manner. During acute infection or vaccination, T cells differentiate into effector cells and later develop into memory cells after antigen clearance, which mediate immune protection against the same antigen. In contrast, during cancer and chronic infection, T cells fail to enter the canonical effector or memory cell differentiation path. Instead, antigen-specific T cells enter a dysfunctional, partially responsive state called exhaustion. Exhausted T cells are heterogeneous. A subset of exhausted T cells exhibits stem cell-like properties. These stem-like T cells sustain immunity through self-renewal and repopulation of terminally differentiated progenies. Stem-like properties are critical for T cell immunity induced by immunotherapy. This review summarizes recent advances in understanding the molecular mechanisms controlling the exhaustion and stemness of T cells and explores the potential of rewiring these circuits to increase the efficiency of T-cell-based immunotherapy.

## Introduction

The differentiation trajectory of CD8 T cells is dictated by the duration of antigen stimulation. During acute infection, naive T cells (_TNs_) differentiate into either effector T cells (_TEFFs_) to clear antigens or memory precursor cells (_TMPs_) [1, 2]. After the antigen is cleared, terminally differentiated short-lived T_EFF_ cells go through a contraction phase to avoid immune pathology, while T_MP_ cells develop into memory T cells (_TMEMs_) to provide a self-renewing antigen-specific T cell pool for long-term immune protection against potential reinfection [1, 2]. Human T_MEM_ cells contain various subsets, including central memory (T_CM_), stem-like memory (T_SCM_), effector memory (T_EM_), and CD45RA^+^ effector memory (T_EMRA_) T cells, that circulate throughout the body and maintain immune memory [3, 4]. Although both T_CM_ and T_SCM_ cells can self-renew, the T_SCM_ population has greater proliferation capacity and multipotency and displays superior antitumor immunity during adoptive cell therapy [4–7]. In addition to the circulating T_MEM_ subsets, a distinct noncirculating tissue-resident memory T cell population (T_RM_) that mediates local immune protection has been described [8–11].

During cancer and chronic infection, antigen-specific CD8 T cells undergo constant TCR stimulation in an immunosuppressive environment, which drives T cells to enter a dysfunctional state called exhaustion [12]. Exhaustion prevents T cells from eradicating infected cells or cancer cells [13–21]. T-cell exhaustion has been characterized in landmark studies in a mouse model of chronic lymphocytic choriomeningitis virus (LCMV) clone 13 infection and has been observed in humans with chronic HIV, HBV, and HCV infections and cancers [13–21]. Exhausted CD8 T (T_EX_) cells progressively lose their effector function, upregulate inhibitory receptors (also termed immune checkpoints), fail to persist or form memory, and become metabolically dysregulated [19–21]. Immune checkpoints, such as CTLA-4, PD-1, LAG-3, TIM3, and TIGIT, transduce inhibitory signals to suppress T cell responses. By blocking these signals, immune checkpoint blockade (ICB) reinvigorates T cell responses. In addition to constant TCR stimulation, interactions with suppressive cells, including myeloid cells, in the tumor microenvironment promote T-cell exhaustion through immune checkpoints, including TIGIT [22]. The epigenetic program of T cell exhaustion is largely unaffected by checkpoint blockade and drives re-exhaustion after cessation of PD-1 blockade [23, 24]. These “epigenetic scars” are characterized by the maintenance of open chromatin at genes associated with T-cell exhaustion after elimination of chronic antigen stimulation [25, 26], suggesting that at least part of the epigenetic program associated with T-cell exhaustion is irreversible once it is established.

In autoimmune diseases, T-cell exhaustion restrains excessive immune activation and is paradoxically associated with favorable clinical outcomes, in contrast to its detrimental role during chronic infection or cancer. Transcriptomic analyses of CD8 T cells from patients with autoimmune disorders such as antineutrophil cytoplasmic antibody-associated vasculitis, systemic lupus erythematosus, and type 1 diabetes revealed that a gene expression signature resembling that of exhausted CD8 T cells in chronic viral infection correlated with reduced relapse frequency and sustained remission [27, 28]. T-cell exhaustion in autoimmune diseases arises from persistent stimulation by autoantigens combined with insufficient CD4 T-cell help [28, 29]. Exhaustion limits immunopathology by dampening autoreactive CD8 T-cell responses, acting as a form of peripheral tolerance once self-reactivity is established [27, 30]. Indeed, genetic or pharmacologic blockade of PD-1 or LAG-3 signaling in mice promotes autoimmune diseases, underscoring the protective role of exhaustion-associated immune checkpoint pathways [29]. Conversely, therapeutic induction of exhaustion, for example, by enhancing PD-1 signaling, has been proposed as a means to mitigate autoimmunity [30]. In organ transplantation, T-cell exhaustion suppresses alloimmune activation and thereby promotes graft tolerance. Persistent alloantigen exposure induces exhausted CD8 T cells with reduced cytokine production, facilitating long-term transplant acceptance [31]. Conversely, disruption of PD-L1–mediated inhibitory signaling enhances T-cell activation and accelerates cardiac allograft rejection and vasculopathy [32]. Clinically, PD-1/PD-L1 blockade restores antitumor responses but often promotes graft rejection, suggesting that maintaining T-cell exhaustion is crucial for sustaining transplant tolerance [33]. Collectively, T-cell exhaustion limits antiviral and antitumor immunity but is beneficial for preventing chronic autoreactivity or alloreactivity.

## Heterogeneity within the T_EX_ lineage

**Fig. 1:**
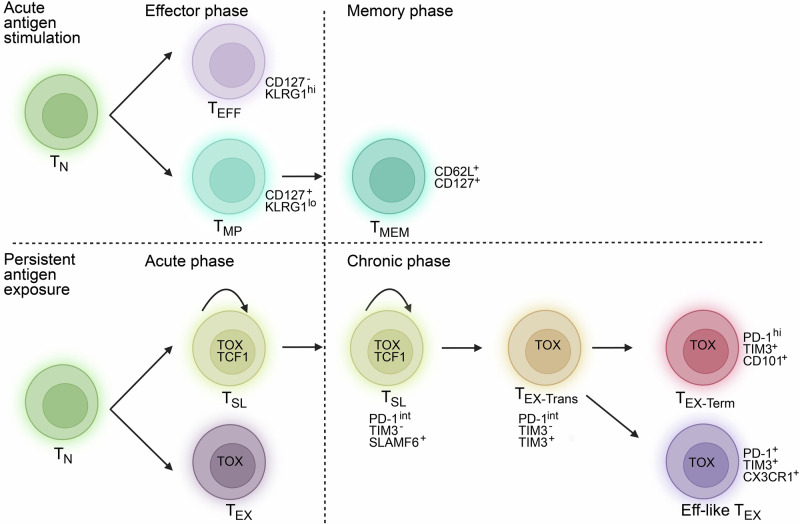
Differentiation trajectories of CD8 T cells during acute and chronic antigen exposure. During acute infection, naïve T cells (_TNs_) differentiate into effector cells that mediate rapid pathogen clearance and memory cells that provide long-term protection. In contrast, persistent antigen stimulation during chronic viral infection or cancer drives an alternative pathway. Early in the immune response, terminal effector-like cells emerge from naïve precursors but decline quickly and show limited persistence. In parallel, stem-like T (TSL) cells, which serve as progenitors of the exhausted lineage, increase and retain self-renewal capacity. T_SL_ cells differentiate into transitory exhausted cells (T_EX-Trans_), which can transiently expand but ultimately progress irreversibly into terminally exhausted cells (T_EX-term_) with fixed dysfunction or into effector-like exhausted cells (eff-like T_EX_)

Like the T_EFF_ and T_MEM_ cells, T_EX_ cells are also heterogeneous (Fig. 1). An early study revealed that a subset of T_EX_ cells is more responsive to PD-1 blockade [34]. Transcription factors (TFs), such as T-BET and EOMES, and surface proteins, such as CD39, have been used to identify T_EX_ cells with progenitor-like or terminally exhausted phenotypes [35, 36]. Perhaps one of the most exciting developments in the field of T_EX_ biology is the discovery of a stem cell-like T cell population that expresses the TF TCF1 during chronic infection and cancer [37–47]. Stem-like T cells (_TSLs_), also termed exhausted progenitor CD8 T cells or T_PEX_, are critical for long-term cellular immunity. To maintain long-term control over chronic infection and cancer, T_SL_ cells self-renew and replenish other exhausted TCF1^−^ populations [37–47]. Stem-like T cells are maintained by conventional type 1 dendritic cells in their niches in the lymph node and tumor stroma, which serve as reservoirs for antitumor T cells in cancer or antiviral T cells during chronic infection [38–40, 48–55]. Compared with their TCF1^−^ counterparts, T_SL_ cells exhibit a superior ability to proliferate in response to immunotherapies, including PD-1 blockade and adoptive cell therapy [37–39, 41, 42, 45, 46, 56–58]. In addition, T_SL_ cells are endowed with greater mitochondrial fitness, which is critical for tumor control [56, 58–60]. The frequency of TCF1^+^CD8 T cells in cancer patients treated with checkpoint inhibitors is associated with favorable clinical outcomes [42, 61, 62]. In addition, the gene signatures of T-cell stemness and/or T-cell memory in premanufactured T cells and in chimeric antigen receptor (CAR) T-cell infusion products positively correlate with the response to CAR-T-cell therapy in cancer patients [63–65]. These studies suggest that the properties of T_SL_ cells are ideal for eliciting optimal T-cell immunity by immunotherapy. Notably, in autoimmune diseases, T_SL_ cells sustain autoreactivity and tissue destruction [66–68].

When and how T_EX_ cells diverge from the differentiation trajectory of T_EFF_ ->T_MEM_ are under active investigation. Rather than being imprinted to become T_EFF_ or T_EX_ during priming, antigen-specific T cells are continuously adapting to the antigenic environment [69]. Recent developments in single-cell omics profiling technologies have enabled us to pinpoint the bifurcation point of the two distinct cell fates. In LCMV infection, single-cell RNA sequencing (scRNA-seq) of CD8 T cells responding to acute infection versus chronic infection diverged during the late stage of initial clonal expansion [44]. Notably, despite the similarities between T_SL_ cells and T_MP_ cells, the TF TOX is expressed only by T_SL_ cells [44]. TOX is essential for the development and persistence of the T_EX_ lineage, including T_SL_ cells, whereas the loss of TOX favors the fate of T_EFF_ but ultimately impairs the persistence of antigen-specific T cells under chronic antigen stimulation [44, 70–74]. Recent discoveries of the common progenitors of T_SL_ cells and T_MP_ cells have revealed the flexibility of early cell fate decisions in both memory formation and exhaustion progression [75, 76].

More recent studies revealed further heterogeneity among T_SL_ or T_PEX_ cells. The expression of CD69 divides these cells into two subsets: a CD69^+^ lymphoid tissue-resident subset (T_EX-Prog1_) and a CD69^−^ T_EX-Prog2_ subset that downregulates TCF1 and enters the blood [77]. Notably, single-cell ATAC+RNA-sequencing analysis of CAR-T cells revealed a T_SL_ subset that shows greater activity of T-box TFs, including EOMES, and may represent a transitory state between the T_SL_ and its progeny [78]. The proliferative potential and multipotency are not evenly distributed among T_SL_ cells. In chronic LCMV infection, a subset of TF-MYB-dependent CD62L^+^ cells within T_SL_ cells retain the highest level of stemness [79]. Importantly, the capacity for long-term self-renewal and a proliferative burst in response to PD-1 blockade are selectively preserved in this small subset of T_SL_ cells [79]. Similarly, T_SL_ cells exhibit a hierarchical distribution of stemness characteristics in cancer. Compared with their TCF1^+^TOX^+^ counterparts, a TCF1^+^TOX^−^ subset in the draining lymph node of the tumor is protected from the epigenetic scar of exhaustion and demonstrates superior antitumor immunity in adoptive cell therapy and PD-1 blockade [51]. Two T_SCM_ subsets, a functional progenitor subset lacking expression of inhibitory receptors and a PD-1^+^TIGIT^+^ exhausted-like subset, are found in human T cells [80].

The progenies of T_SL_ cells are also heterogeneous. T_SL_ cells first differentiate into CD101^−^TIM3^+^ transitory T_EX_ cells, which exhibit partial effector function and respond to PD-1 blockade [81]. The transitory subset then differentiates into the terminally exhausted CD101^+^TIM3^+^ subset (T_EX-Term_) [81]. Compared with intermediate T_EX_ cells, T_EX-Term_ cells upregulate CD69 expression [77]. scRNA-seq revealed another potential differentiation pattern in which the T_SL_ subset bifurcates into two distinct progenies, the T_EX-Term_ subset and an IL-21-dependent KLRG1^+^CX3CR1^+^ subset that exhibits superior effector function [47, 82]. In chronic LCMV infection, CX3CR1^+^ eff-like T_EX_ cells are closer to the circulation, whereas CXCR6^+^CX3CR1^−^ T_EX-Term_ cells reside in tissues [83]. Because of its short-lived nature, the eff-like T_EX_ subset needs to be continuously replenished by TCF1^+^ T_SL_ cells [47]. In cancer patients, CX3CR1 is expressed in a CD8 T cell population that responds to chemoimmunotherapy [84]. The recent development of single-cell multiomics provides further insight into the diversity within the T_EX-Term_ and eff-like T_EX_ subsets [85, 86]. Notably, CXCR6 is required to position eff-like T_EX_ cells in proximity to CCR7^+^ conventional DCs that trans-present IL15 to facilitate the survival of T cells [87]. Thus, CXCR6 itself may not drive T cell exhaustion.

## The molecular circuit regulating the exhaustion and stemness of T cells

### The transcriptional program of T cell exhaustion

**Fig. 2:**
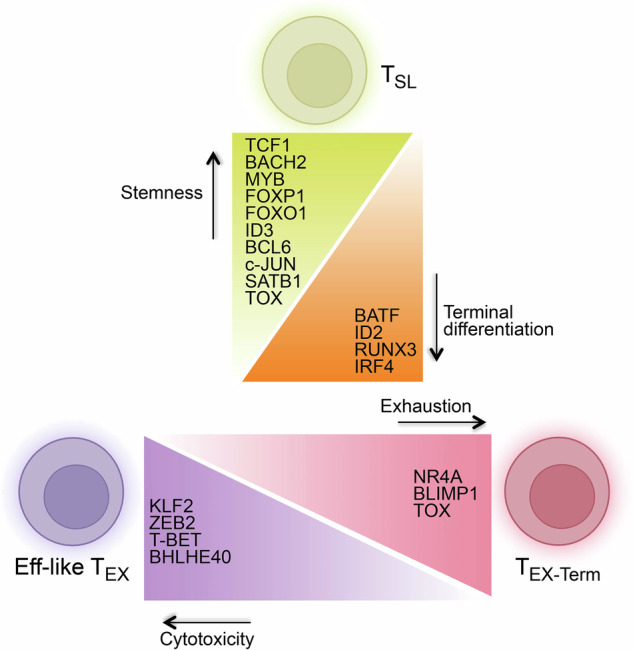
Transcriptional regulators of T_SL_, eff-like T_EX_, and T_EX-term_. Development and/or maintenance of T_SL_ is driven by transcription factors such as TCF1, BACH2, MYB, FOXP1, FOXO1, ID3, BCL6, c-JUN, and SATB1. The transcription factors BATF, IRF4, ID2, and RUNX3 promote the terminal differentiation of CD8 T cells and suppress stem-like cell fate. Among the terminally differentiated subsets, the eff-like T_EX_ subset is positively regulated by KLF2, ZEB2, T-BET, and BHLHE40, whereas NR4A and BLIMP1 promote terminal exhaustion. Both the T_SL_ and T_EX_-Term lineages require TOX

While many transcriptional regulatory circuits are shared between acute and chronic antigen exposure, some transcriptional signaling cascades are specific to the adaptation of T_EX_ subsets to chronic antigen stimulation (Fig. 2). The typical “exhaustion-specific” TF is TOX, which defines the T_EX_ lineage and plays key roles in all exhausted T cell subset differentiation processes [44, 70–74]. High expression of TOX is a direct consequence of strong and constant TCR stimulation, and it can be a potential adaptive mechanism to maintain CD8 T cell internal homeostasis at the transcriptional and epigenetic level [44, 70–74]. Indeed, overexpression of TOX improves the persistence of virus-specific CD8 T cells during chronic infection [44], suggesting that TOX plays a beneficial role in the adaptation of T cells to chronic stimulation. Removing TOX drives the differentiation of antigen-specific CD8 T cells to short-lived T_EFF_ cells while impairing the differentiation of the T_SL_ population [44, 70–74]. Consequently, in the long term, all T_EX_ cell subsets fail to persist [44, 70–72]. In addition to TCR signaling, LAG-3 also sustains TOX to facilitate the development of T_EX_ lineages [88]. T_SL_ cells are maintained by sustained TCR stimulation [55]. Cessation of TCR signaling prompts T_SL_ cells to diverge to a T_MEM_ fate, which is accompanied by downregulation of TOX [75, 76, 89]. These findings are consistent with the notion that TOX is a key feature that distinguishes T_SL_ cells from T_MP_ cells [44]. Notably, the effect of TOX may be dosage dependent. A partial reduction in TOX levels results in effective tumor control without compromising long-term T cell immunity [70]. Other TFs critical for the development of T cell exhaustion, such as NR4A family members [73, 90–92], NFAT-AP-1 signaling [93–96], and BATF/IRF4 [97–100], are also associated with TCR signaling.

### TCF1– the “identity” of stemness

TCF1, the most broadly reported pro-stem TF, has two major isoforms (short p33 and long p45) with distinct functions in different biological processes [101]. TCF1 was first reported as a master regulator during T cell development in the thymus via Notch signaling [102, 103]. As a pioneer factor, TCF1 has the capacity to shape and reprogram the epigenetic landscape toward a functional mature T cell stage to initiate T cell identity [104]. Together with its homolog LEF1, TCF1 establishes the epigenetic landscape of T cells by controlling both histone acetylation and chromatin architecture via the intrinsic HDAC activity of its short isoform p33 [105] and by coordinating with CTCF [106]. Thus, from a developmental perspective, TCF1 has the capacity to modulate T cell identity toward a naive mature T cell stage and maintain T cell stemness with strong epigenetic footprints.

During acute antigen exposure, TCF1 restrains hyper-effector proliferation and modulates the memory T cell pool to maintain its capacity for secondary responses [6, 107–109]. A lack of TCF1 has a limited effect on the initial immune response; however, it has a significantly strong effect on recall toward the same antigen, suggesting that it plays a key role in maintaining the stemness of memory T cells [107–109]. In particular, the TCF1 p45 isoform contributes to optimal memory formation [110]. TCF1 has been shown to regulate multiple downstream pathways contributing to memory formation, including EOMES and BCL-2 [107, 108]. Interestingly, moderate levels of coinhibitory signaling molecules such as PD-1 and LAG-3 during acute infection help T cells maintain memory capacity and high expression of TCF1. In fact, intermediate levels of PD-1 expression marked a T_SCM_ population with high CD62L expression, high self-renewal capacity with secondary transfers, and, most importantly, better genomic protection [5].

During chronic infection, TCF1 acts as a master regulator and maintains a pool of T_SL_ cells that renew themselves even in the presence of constant antigen stimulation and mount a proliferative burst to ICB. In the LCMV chronic infection model, TCF1 was first defined as the core TF that regulates a follicular-like CD8 T cell population with a major capacity for self-renewal during chronic infection and response to PD-1 blockade [37–41], similar to the regulatory circuitry of follicular helper T cells (TFHs) [111]. This TCF1^+^ population is defined as progenitors for exhausted T cells during chronic infection and is negatively associated with IFN signaling sensing [37, 112]. The major follicular features of T_SL_ cells are that they are CXCR5^+^ and Ly108^+^ and are exclusive to terminal differentiation markers such as TIM3 and CD39 [37–39, 47, 82]. In the early stages of chronic infection, TCF1 restrains terminal differentiation and promotes T_SL_ generation [47]. At the late stage of chronic infection, TCF1 is required for the persistence of the antigen-specific CD8 T cell population, including the T_EX-Term_ and eff-like T_EX_ subsets, which can be either binarily differentiated from T_SL_ cells [81, 82] or from a tissue-circulation-tissue-migration manner for peripheral proliferation of antigen-specific T cells [77, 113]. Notably, similar to the acute setting, the TCF1 isoform p45 plays a strong role in maintaining T_SL_ cell fate [47], suggesting a potential regulatory role of Wnt-β-catenin signaling in regulating T_SL_ cell identity maintenance [6].

### Master regulators of stem-like T cells and their progenies

In addition to TCF1, multiple TFs are involved in maintaining T cell stemness during either acute or chronic infection and targeting different signaling cascades, such as those involved in T cell quiescence, survival, metabolism, and transcription.

BACH2 maintains the naïve differentiation state of mature T cells and is critical for the development of T_CM_ and regulatory T cells [114–118]. This effect of BACH2 further extends as a major factor in repressing the terminal exhaustion program and maintaining the transcriptional and epigenetic landscape of T_SL_ cells during both acute and chronic infection [119, 120]. Transcriptionally, BACH2 has a shared motif comparable to that of AP-1, and the expression/motif usage of BACH2 and AP-1 strongly antagonizes both mouse and human T cell differentiation trajectories [116, 119, 120]. This potential transcriptional competition between BACH2 and AP-1 occurs on the enhancer of TCR-induced activation genes, and BACH2 reshapes the whole epigenetic landscape of CD8 T cells toward a more stemness-restrained pattern [116, 119, 120]. Notably, the expression level of BACH2 is correlated with the degree of stemness in CAR-T cells and can be harnessed to optimize the antitumor immunity of CAR-T cells [121]. In addition to AP-1 TFs, BACH2 also antagonizes RUNX3 and BLIMP1, both of which are important players in the terminal differentiation and exhaustion of T cells [119].

MYB has been reported to be among the major regulators of hematopoietic stem cell maintenance and lymphocyte lineage commitment [122–124]. It is also directly involved in regulating T cell development at different stages [125, 126], suggesting that it may have a role similar to that of TCF1 in reshaping the epigenetic landscape of “naive-like” T cells. During acute infection, MYB is strongly enriched in memory-associated populations, particularly in the CD62L^+^ T_SCM_ population, and is the central factor that maintains memory cell homeostasis [127, 128]. Manipulating MYB strongly affects memory recall responses and polyfunctionality. For example, enhancing MYB expression results in better memory T cell survival via increased BCL-2/BCL-XL expression and durable polyfunctional responses of multiple cytokines via potential oxidative metabolic processes [127, 128]. This MYB-driven enhanced stemness signaling cascade is also associated with an increased CD62L^+^ T_PEX_ population during chronic infection, which further strongly contributes to T_PEX_ population survival as well as the response to PD-1 blockade [47, 79]. MYB overexpression in therapeutic T cells enhances tumor control during adoptive cell therapy [128].

FOXO1, a major TF that is involved in multiple metabolic processes to increase gluconeogenesis [129], also plays a key role in stemness maintenance in T_N_ and T_CM_ cell populations [130, 131]. FOXO1 generally enhances memory T cell formation via the inhibition of effector-associated genes such as T-bet [132] and promotes the expression of memory-associated receptors such as IL7RA and CCR7, even at the early stage of activation [133, 134]. During chronic infection, FOXO1 is a key regulator that maintains PD-1 expression and ensures the survival of the T_SL_ population [135, 136]. This FOXO1-PD-1 axis further influences metabolic regulation, where the FOXO1-PGC1a pathway counterbalances PD-1-driven exhaustion and maintains partial functionality of antigen-responsive T cells [137].

FOXP1 is a member of the Forkhead box (FOX) TF family. In T cells, FOXP1 was first described as a TF that enforces T cell quiescence and suppresses FOXO1 and the MEK/ERK pathway [138]. Deletion of FOXP1 in naïve T cells induces an effector-like phenotype in lymphopenic mice [138]. FOXP1 is also required for the homeostasis and suppressive function of regulatory T cells [139, 140]. CD8 T cells from mice in which FOXP1 is deleted from T cells during development exhibit increased effector function and antitumor immunity [141]. Surprisingly, acutely disrupting FOXP1 in CD8^+^ CAR-T cells impaired expansion and tumor control by CAR-T cells [78]. In addition, FOXP1 deficiency compromises the differentiation of T_SL_ cells and promotes the premature transition from T_SL_ to T_EFF_ CAR-T cells [78]. Mechanistically, FOXP1 deficiency increases chromatin accessibility to TCR downstream TFs, including AP-1 and NR4A family TFs. Thus, FOXP1 may play context-dependent roles at different stages of T cell differentiation. Future studies are warranted to determine how to optimize T cell immunity during immunotherapy by harnessing the activity of FOXP1.

The zinc finger TF KLF2 is best known as a master regulator that promotes T cell egress from lymphoid tissues and regulates the expression of S1PR1, CD62L, and integrin β7 [142]. KLF2, which is highly expressed in naïve T cells, is a gatekeeper for T cell activation and restrains cytokine production [143]. The downregulation of KLF2 and S1PR1 is required for the formation of T_RM_ cells [144]. T_SL_ and T_EFF_ cells are predominantly found in CAR-T cells recovered from hosts that have cleared tumors [78]. Simultaneous profiling of the single-cell transcriptome and epigenome established the gene regulatory network of T_EFF_-like CAR-T cells and revealed that KLF2 is a hub TF [78]. KLF2-deficient CAR-T cells exhibit profound defects in the generation of the T_EFF_-like subset [78]. Instead, KLF2-deficient CAR-T cells display an exhaustion-like phenotype and upregulate the expression of inhibitory receptors and TOX [78]. KLF2 deficiency also downregulates effector molecules and impairs in vitro killing by T cells [78]. In mice with solid tumors, KLF2 deficiency reduces tumor infiltration by CAR-T cells [78]. Single-cell epigenetic analysis revealed that KLF2 deficiency increases chromatin accessibility at binding sites of AP-1 and NFAT TFs while decreasing chromatin accessibility at binding sites of KLF and T-box TFs [78]. Like CAR-T cells, virus-specific CD8 T cells that respond to acute LCMV or MHV infection also exhibit marked defects in T_EFF_ differentiation and upregulation of the exhaustion signature [145, 146]. Thus, KLF2 may represent a master switch controlling the cell fate decision between T_EFF_ and T_EX_ lineages. It is worth further investigating whether the effect of KLF2 on T_EFF_ versus T_EX_ differentiation is connected to its role in regulating T cell migration. In addition, whether targeting KLF2 activity in therapeutic T cells improves their synergy with ICB awaits further investigation.

### Transcriptional circuits determine T cell effectiveness versus persistence

While these major TFs individually contribute to T cell stemness, they also form a transcriptional network core in which these TFs maintain the expression level of one another. For example, TCF1 is critical for maintaining the expression of MYB; however, alteration of the expression level of TCF1 is among the major phenotypes of MYB genetic perturbation [47, 79]. While FOXO1 may coordinate with TCF1 and BACH2 to promote stemness at the epigenetic level [119, 147, 148], both TCF1 and BACH2 can be potential direct targets of FOXO1 [134]. These TFs inside the stemness core self-enhance the expression and function of each other to restrain T cells in a high-proliferative capacity but low-activation stage. Removal of these TF core candidates usually results in a limited or no reduction in T_EFF_ populations but has a significant effect on the development of T_SL_ cells during chronic antigen stimulation.

Another important feature of the function of this stemness TF core is that multiple candidates can respond to the same upstream signals. For example, an intermediate level of PD-1 expression is important for maintaining the expression of both TCF1 and FOXO1 [5, 47, 135, 137], whereas type 1 interferon signaling inhibits both of them [37, 149]. MYB and FOXO1 share the same upstream miRNA, miR-150, to inhibit their expression [127, 150]. These coordinated upstream signals can upregulate or downregulate TFs in this stemness transcriptional core to make the cell fate decide whether to maintain commitment to a stem-like state or terminally differentiate in response to an antigen.

In addition to the core module, multiple polarized TF pairs affect “effectiveness” versus “persistence” during CD8 T cell responses. These TF pairs include T-BET versus EOMES [35, 151], ID2 versus ID3 [152, 153], BLIMP1 versus BCL6 [154, 155], STAT4 versus STAT3 [156–158], and ZEB2 versus ZEB1 [159, 160]. Most of these TF pairs have the feature of tuning the same functional module with different activation intensities. For example, both T-bet and Eomes are T-box family members that can drive the expression of *Ifng* and other effector genes. T-bet, however, has much stronger functionality in pushing cells into an “effective” module and overactivation via T-bet-triggered terminal Teff differentiation [2], whereas Eomes maintains a partial response capacity to the antigens in both post-Teff contraction during acute infection and cellular persistence during chronic infection and cancer progression [161–163]. Similar rationales apply to Blimp1 versus Bcl6 [154], although Blimp1^+^ cells are considered to be more terminal CD8 T cells with high cytotoxicity but limited cytokine-secreting capacity [164–167].

TFs involved in stem cell maintenance primarily function by promoting “persistence-biased” TFs to retain the stem-like or progenitor identity of CD8 T cells or by directly inhibiting “effectiveness-biased” TFs. TCF1, FOXO1, and MYB are known to promote Eomes expression and mediate the T-bet-to-Eomes transition after the Teff boost phase, both in acute and chronic infections [47, 168]. TCF1 also enhances and maintains the expression of *Bcl6* and *Id3* to promote T_MP_ or T_SL_ cell fate, and the latter TFs drive a function-specific molecular module to ensure T cell persistence [37, 39, 40, 169, 170]. The chromatin organizer SATB1 maintains the quiescent and stem-like state of T_SL_ cells and inhibits expansion and effector differentiation during chronic infection and cancer by regulating transcriptional programs, chromatin accessibility, and genome architecture at key stemness-associated loci such as *Tcf7*, *Bach2*, and *Myb* [171, 172].

Recently, researchers have discovered the functions of more transcriptional circuits that are related to a “persistence-to-terminal effectiveness” transition rationale. For example, a study revealed that the ETS family member Fli1 is a transcriptional immune checkpoint that inhibits hyper-Teff responses in both multiple infection and cancer models. Fli1 directly inhibits the *cis*-regulatory elements on effector-associated genes by competing with Runx3 [173], which drives T_EFF_ responses, particularly in pathological tissue [174–177]. Furthermore, in addition to these transcriptional checkpoints, several TFs previously known to regulate T_H_2 versus T_H_1 responses, including GATA3 and EGR2, also promote “naiveness” or “stemness” modules during CD8 T cell responses. GATA3 inhibits Teff differentiation, potentially by suppressing the expression of the terminal Teff TF BHLHE40 [178–180], whereas EGR2 contributes to the expression of multiple persistence module TFs, including FOXO1 and Eomes [181].

### Metabolic adaptation of T_EX_ cells to chronic antigen stimulation

Metabolism is a critical determinant of T-cell function. The exchange of metabolites between T cells and their surrounding environment profoundly influences T cell fate. Dysregulation of cellular energy metabolism in exhausted T cells not only limits their bioenergetic capacity but also reshapes their epigenetic program.

Exhausted CD8 T cells in chronic infections and tumors exhibit marked impairment in core bioenergetic pathways, with both glycolysis and oxidative phosphorylation (OXPHOS) substantially reduced [137, 182–185]. Mitochondria in exhausted T cells display reduced mass, lower membrane potential, and impaired respiratory reserve, changes that are closely associated with decreased expression of PGC1α, a central regulator of mitochondrial biogenesis and antioxidant defense [137, 182–185]. Depolarized mitochondria in CD8⁺ tumor-infiltrating lymphocytes (TILs), resulting from impaired mitophagy, drive terminal exhaustion through epigenetic reprogramming. Enhancing mitochondrial fitness with nicotinamide riboside alleviated dysfunction and improved the response to PD-1 blockade [186]. In parallel, glycolytic flux is suppressed through both extrinsic and intrinsic mechanisms [137, 185, 187]. Nutrient limitation in the tumor microenvironment restricts glucose uptake, while persistent signaling through the PD-1 pathway inhibits aerobic glycolysis, further exacerbating metabolic insufficiency [137]. Metabolic reprogramming of T cells by increasing phosphoenolpyruvate production via PCK1 overexpression enhances effector function and tumor control by T cells [187]. Together, mitochondrial and glycolytic defects create an energy-deficient state that reinforces functional decline in exhausted T cells.

The metabolic state of exhausted T cells directly shapes their epigenetic landscape. Key metabolites such as acetyl-CoA, α-ketoglutarate, and S-adenosylmethionine (SAM) act as substrates or cofactors for histone acetylation and methylation, thereby modulating gene expression profiles that are central to T-cell fate [188–192]. Perturbations in amino acid metabolism can also have lasting epigenetic consequences. Methionine availability regulates methyl group donation for histone and DNA methylation, whereas tryptophan catabolism alters chromatin states [188, 193–195]. Metabolic–epigenetic coupling stabilizes exhaustion-associated programs, making T cells resistant to functional reprogramming even when inhibitory receptor signaling is blocked.

Beyond mitochondrial bioenergetics and metabolic–epigenetic coupling, additional metabolites and nutrient pathways critically influence the establishment and persistence of T-cell exhaustion. In the tumor microenvironment, the depletion of amino acids such as arginine and serine impairs proliferation, cytokine secretion, and receptor expression [196, 197]. Dysregulated lipid metabolism is a common feature of PD-1^hi^ TILs and is characterized by the accumulation of cholesterol and fatty acids [198, 199]. These lipid deposits induce endoplasmic reticulum stress, thereby impairing effector T-cell function. However, cholesterol deficiency also impairs the effector function of tumor-infiltrating T cells [200]. Conjugated bile acids accumulate in liver cancer, whereas inhibiting their synthesis improves T cell function and sensitivity to ICB [201]. Hypoxia has dual effects on T cells. While HIF signaling promotes glycolytic metabolism and augments effector activity in certain contexts [202–204], it may simultaneously induce inhibitory receptor expression and dampen cytotoxic function [205]. Importantly, under persistent antigenic stimulation, hypoxic stress accelerates this dysfunction by enforcing Blimp1–mediated repression of PGC1α-dependent mitochondrial reprogramming [206, 207]. Other metabolic by-products regulate T-cell exhaustion and differentiation. Succinate, a TCA cycle metabolite that accumulates in SDH-deficient tumors, enhances CD8 T cell stemness and persistence through mitochondrial and epigenetic remodeling and thereby improves the response to CAR-T and checkpoint blockade therapies [208]. Acidic metabolic waste accumulated in the tumor microenvironment paradoxically preserved T cell stemness and enhanced persistent antitumor T-cell immunity [209]. Clearance of ammonia, a byproduct of amino acid metabolism, is required for the development of T cell memory and can be targeted to improve adoptive cell therapy [210]. Additional by-products, such as tumor-derived lactate and excess extracellular potassium, also regulate exhaustion by directly impairing effector function or skewing differentiation toward stem-like states [211–213]. Notably, stiffness of the extracellular matrix is a hallmark of cancer and promotes exhaustion through the PIEZO1-OSR2 axis [214]. Thus, T-cell exhaustion is not caused by a single metabolic defect but by a complex interplay of nutrient availability, metabolic activity, and environmental stressors.

TCR activation triggers calcium release from the endoplasmic reticulum [215]. Increased cytosolic calcium is subsequently taken up by mitochondria, which are the primary sites of oxidative phosphorylation, and increases the activity of multiple TCA cycle enzymes. This increase in enzymatic activity promotes the generation of redox cofactors and increases reactive oxygen species (ROS) production. In both tumor and chronic infection models, persistent antigenic stimulation drives mitochondrial dysfunction in T cells, leading to impaired oxidative phosphorylation, ATP depletion, and ROS accumulation [216]. These redox-driven defects enforce terminal exhaustion by suppressing self-renewal programs and activating exhaustion-associated TFs, whereas antioxidant treatment restores proliferation, effector function, and progenitor-like features, thereby enhancing antitumor immunity [216]. While excessive ROS are detrimental to the T cell response, ROS also play an important role in T cell activation [217]. KEAP1 is a key sensor of oxidative stress. Under basal conditions, it targets the TF NRF2 for proteasomal degradation. Upon oxidation of reactive cysteine residues, NRF2 is released from KEAP1, which is subsequently translocated to the nucleus, where it activates the expression of antioxidant genes. KEAP1 expression is essential for CD8 T cells to adapt to chronic antigens because it prevents NRF2-driven hyperactivation of TCR signaling, cell death, and metabolic dysregulation [218]. KEAP1 deficiency and NRF2 hyperactivation reduce the T_SL_ subset and lead to the accumulation of T_EX_ with a terminal exhaustion phenotype [218]. NRF2 promotes exhaustion by upregulating the expression of the immune checkpoint PTGIR, which impairs metabolism and cytokine production by T cells [219]. In the context of asparagine restriction, however, NRF2 plays a positive role in the metabolic fitness and antitumor response of T cells [220]. The precise impact of the KEAP1-NRF2 axis on the T cell response may be context dependent.

### Targeting the molecular program of T cell exhaustion and stemness to improve immunotherapy efficacy

Successful T-cell-based cancer immunotherapy depends on the balanced differentiation of T-cell effectiveness and persistence. It has been shown that the TCF1^+^ T_SL_ population in the tumor microenvironment is the major population that responds to ICB, and these cells differentiate into further reinvigorated eff-like T_EX_ cells to eliminate tumor growth [42, 45, 221, 222]. According to multiple scRNA-seq studies of tumor-infiltrated immune cells, the abundance of T_SL_ cells is a prognostic marker for ICB treatment in different cancer types, including melanoma [223], breast cancer [224], and renal cell carcinoma [225]. While this persistent transcriptional module is important for maintaining the antigen-specific cellular response pool to ICBs, the major reinvigoration feature of ICB-treated antigen-specific CD8 T cells is eff-like T_EX_ reactivation [23]. In clinical studies, enhanced T cell response features, such as stronger cell cycling and effector-associated molecule expression, have also been reported to be associated with better outcomes [226, 227], although these effector-like cells share T_EX_ receptor profiles [228–232]. Thus, modulating transcriptional circuits to reinforce T_EFF_-associated responses has also been a working hypothesis in several studies that involved targeting TOX [44, 70–74], Fli1 [173], and Blimp1/NR4A3 [233] or enhancing STAT5 signaling [113].

The transcriptional features of CAR-T cells in the tumor microenvironment are similar to those of infection-model-defined T_EX_ cells [78, 88, 234], with an increase in effector features over time in non-Hodgkin lymphoma patients but an increased AP-1/NR4A/BLIMP1 TF profile in the TIGIT^+^ CAR-T cell population in the nonresponsive group [234]. Similar dysfunctional CAR-T cell features with increased Blimp1/NR4A3 expression were also observed in metastatic prostate cancer treatment, in which targeting these two TFs increased the therapeutic effect of CAR-T cells in murine models [233]. Deletion of the pro-exhaustion TF ETV7 also enhances the antitumor efficacy of CD8 T cells [235]. Furthermore, in vitro CRISPR screening revealed TLE4 and IKZF2 as negative regulators that restrict the effects of effector-like CAR-T cells against glioblastomas [236]. In addition to tuning the effector and terminal exhaustion balance, several other studies have focused on enhancing stem-like differentiation during CAR-T-cell responses and have highlighted the importance of the stemness module during cancer treatment. An earlier study of Listeria monocytogenes infection revealed that deleting the histone H3 lysine 9 methyltransferase Suv39h1 promotes the stemness of T cells [237]. Consistently, disruption of Suv39h1 in CAR-T cells improves stemness, expansion, persistence, and tumor control [238]. In addition to Suv39h1, disrupting other epigenetic regulators, such as DNMT3a, TET2, and ASXL1, also enhances T cell stemness and antitumor T cell immunity in adoptive cell therapy [239, 240] and ICB [241]. The AP-1 TF c-JUN promotes a T_SL_-like phenotype in CAR-T cells [242]. Another AP-1 member, BATF, is the key TF downstream of PD-1 [98] and is involved in early T cell activation [243]. BATF can amplify effector-like T cell features during chronicity [244], and targeting BATF in CAR-T cells enhances the stemness module for a long-term robust response [245]. Notably, the deletion of REGNASE-1, which targets BATF, in T cells programs the long-term antitumor efficacy of adoptive cell therapy [99]. Combined deletion of REGNASE-1 and BCOR synergistically induces an immortal stem-like state and enhances the function of CAR-T cells [246]. Furthermore, while activating more IL2-STAT5 transcriptional circuits may be an important strategy for promoting stronger eff-like T_EX_ signatures [77, 113, 247–249], activating more stemness-related modules via IL10-STAT3 signaling may also be a viable strategy for achieving better therapeutic outcomes [250, 251]. These studies indicate several potential general mechanisms involved in the transcriptional regulation of CAR-T-cell responses: (1) Effector and exhausted T-cells are defined on the basis of the functional capacity per cell, and both of them can be driven into terminal stages via transcriptional circuits involving TFs such as Blimp1; (2) AP-1 activation is a major feature of CAR-T-cell activation; however, different AP-1 family members may trigger different downstream effects in tuning the effector versus stemness modules, potentially by involving different TF co-binders such as IRF4 or NFAT; and (3) considering that BACH2 inhibits broad AP-1 function and locks the cell into a stemness stage, it is important to orchestrate BACH2 and AP-1 levels to maintain the balance in the differentiation of CAR-T-cells in vivo for the best potential outcomes. (4) STAT signaling activation in the tumor microenvironment is among the key factors involved in the differentiation of the T cell response, with STAT5 signaling being more biased toward effector differentiation and STAT3 signaling being more biased toward stemness. Both strategies may benefit clinical outcomes, but in different scenarios.

How should we choose to enhance short-term effector function or stem-like differentiation and the persistence of CD8 T cell responses during disease treatment, particularly in cancer immunotherapies? One major potential prediagnostic identifier is the “effective immune–tumor intensity ratio.” Previous studies have shown that the “responsive T cell-to-tumor” ratio can be a key marker for predicting the clinical outcome of anti-PD-1 responses [252, 253]. Indeed, combining immunotherapy with chemotherapy or radiotherapy enhances the clinical response and has demonstrated potential benefits across a variety of clinical scenarios [254, 255]. In addition to the effects of extraantigen exposure as well as local inflammatory immune microenvironment reorganization [255], one of the potential reasons for better outcomes in some of these situations is increasing the “effective immune–tumor intensity ratio”. These findings indicate that there are two potential outcomes after treatment: (1) Antigen-specific T cells respond strongly to a limited tumor volume. In this context, a stronger effector T cell response is more likely to trigger a favorable clinical outcome, with the capacity to achieve tumor clearance at least at the given lesion level. (2) The antigen-specific T cell population has some response but is not able to clear tumor cells in the lesion in the short term. Under these conditions, the persistence of exhausted T cells is important for maintaining immune–tumor equilibrium and a partial response or stable disease. This notion is supported by a recent study on the ICB-induced T cell response in murine tumors with different levels of immunogenicity [256].

A topic that has recently garnered increased research efforts is whether we can rewire the molecular circuits of T cells to achieve a context-specific T cell response, particularly in the tumor microenvironment. In the past decade, the syn-Notch system has been developed to sequentially arrange tumor microenvironmental signaling activation toward local CAR expression in T cells, thus triggering only an intratumoral CAR-T cell response at target sites [257–259]. Additionally, the development of an orthogonal cytokine system offers the ability to improve cytokine treatment by supporting the persistence of infiltrating T cells and promoting lesion-dependent, local antigen-specific T cell proliferation [113, 260, 261]. Furthermore, the recent development of integrating CAR constructs, particularly into PD-1 loci, suggests the possibility of using exhaustion-specific DNA regulatory elements to achieve tumor microenvironment-specific functional molecular responses [262, 263]. These strategies aim to initiate strong antigen-specific T cell responses in the lesion area while eliminating off-site T cell activation to reduce potential immune-related adverse events in patients. Thus, future efforts may dive deeper into rewiring molecular circuits of the T cell response at the lesion microenvironmental level to achieve precision microenvironmental medicine.

### Druggable targets and interventional modalities

Recent studies have revealed diverse regulatory checkpoints that control T cell exhaustion, many of which represent potential druggable targets for immune modulation. At the epigenetic level, inhibition of the histone demethylase LSD1 by a small-molecule drug preserves the progenitor-exhausted T cell pool and sustains durable responses to PD-1 blockade by counteracting TCF1 repression and terminal differentiation [264]. Despite their critical role in controlling T cell fates, most master transcriptional regulators are challenging to target using traditional small-molecule therapeutics. Emerging strategies such as PROTACs and molecular glues offer promising alternatives for modulating these TFs. Degradation of the nuclear receptor NR4A1, which represses the effector program in exhausted T cells, by the PROTAC NR-V04 reprograms the tumor microenvironment by enhancing the response of effector-memory CD8 T cells and reducing suppressive myeloid populations [265]. A molecular glue targeting IKZF2 rescues exhausted T cells and potentiates immune control of tumors [266]. In addition, chemical switches can be fused to TFs such as BACH2 to exert temporal and tunable control of T cell differentiation and improve the efficacy of CAR-T-cell therapy [121]. A screen of chromatin-modifying drugs revealed HDAC inhibitors that increase the persistence and repress the exhaustion of CAR-T cells to promote their antitumor immunity [267]. HDAC inhibitors also synergize with PD-1 blockade to enhance antitumor T cell responses [268]. Inhibitors of the EZH2 protein, a core component of the polycomb repressive complex 2, increase CAR-T-cell efficacy by directly repressing exhaustion [269]. JQ1, a small-molecule inhibitor of BRD4, enhances the persistence and antitumor immunity of T cells in adoptive cell therapy while preventing terminal differentiation [270, 271]. The in vitro manufacturing of therapeutic T cells, including CAR-T cells, offers a unique opportunity to rewire their cellular programs with chemical treatments while avoiding direct drug exposure in patients. Treatment with ibrutinib, which inhibits ITK and BTK, during manufacturing promotes survival and stemness and represses the exhaustion of CAR-T cells [218, 272]. Pretreatment of CAR-T cells with inhibitors targeting AKT or MEK attenuated exhaustion and terminal differentiation and potentiated the antitumor efficacy of CAR-T cells in vivo [273, 274]. Interestingly, lithium carbonate treatment enhances antitumor immunity in T cells through directing lactate to mitochondria and could improve T cell-based immunotherapy [275]. Therefore, targeting key regulators of T cell exhaustion and stemness, either in vivo or in vitro, constitutes a viable strategy to improve the antitumor efficacy of T cells.

In this article, we summarize the major molecular circuits that regulate the stemness and exhaustion of T cells and the differentiation of T_SL_ cells into different T_EX_ progenies. We further discuss how the balance between the short-term response to T_EFF_ cells and long-term persistence sustained by T_SL_ cells affects the outcome of T cell-mediated immune responses in cancer and chronic infection. Finally, we discuss the current status and future directions for harnessing molecular circuits to control T cell differentiation in T-cell-based immunotherapy.
